# Integrase Interactor 1 (INI-1) Deficient Renal Cell Carcinoma

**DOI:** 10.7759/cureus.13082

**Published:** 2021-02-02

**Authors:** Manpreet Singh, Harkirat Singh, Benjamin Hambro, Jasleen Kaur, Ravi Rao

**Affiliations:** 1 Internal Medicine, St Agnes Medical Center, Fresno, USA; 2 Hematology and Oncology, St Agnes Medical Center, Fresno, USA

**Keywords:** renal medullary carcinoma, ini-1, renal cell carcinoma, swi/snf, smarcb1, integrase interactor 1

## Abstract

Members of the SWItch/sucrose nonfermentable (SWI-SNF) family, including SWI/SNF related, matrix-associated, actin-dependent regulator of chromatin, subfamily A, member 4 (SMARCA4), SWI/SNF related, matrix‐associated, actin‐dependent regulator of chromatin, subfamily B member 1 (SMARCB1)/integrase interactor 1 (INI-1) are known tumor suppressor genes. Interactions between SMARCB1/INI-1 and key protein components in various cellular pathways are related to tumor progression and proliferation.SMARCB1/INI-1 protein was undetectable in rhabdoid tumor cells, whereas non-tumorous cells express the SMARCB1/INI-1 genes. Germline and sporadic mutations of several genes encoding for proteins in this complex are known to cause a spectrum of cancers, usually with sarcomatoid features which include a very aggressive renal medullary carcinoma. We report a case of a 29-year-old male who presented with SMARCA4 deficient renal tumor with a very aggressive clinical behavior which ultimately led to his death.

## Introduction

Renal medullary carcinoma is a rare and very aggressive malignancy affecting young adults with rare cases in patients with sickle cell disease or trait [1]. The tumor arises predominantly in the renal medulla and exhibits a variety of growth patterns including reticular, solid, tubular, trabecular, cribriform, sarcomatoid, and micropapillary [1]. Fatality rate can approach near 100% in cases within weeks to months after diagnosis. The tumor-suppressor gene SWItch/sucrose nonfermentable (SWI-SNF), which includes SWI/SNF related, matrix-associated, actin-dependent regulator of chromatin, subfamily A, member 4 (SMARCA4) and SWI/SNF related, matrix‐associated, actin‐dependent regulator of chromatin, subfamily B member 1 (SMARCB1)/integrase interactor 1 (INI-1) was first studied in malignant rhabdoid tumors (MRT) of infancy [2]. Mammalian SWI/SNF’s consist of 12-15 subunits, of which an adenosine triphosphatase (ATPase) subunit [either SMARCA2 (BRM) or SMARCA4 (BRG1)] and three additional subunits [SMARCB1 (INI1), SMARCC1, SMARCC2] form the functional core [3]. The complex has been studied to perform a variety of chromatin functions consisting of repositioning, ejecting, and exchanging nucleosomes, which at the cellular process aims to assist with transcription, cell cycle control, proliferation, differentiation, and repair of DNA lesions [3]. MRT can occur in the kidney, central nervous system, and extracranial/extrarenal locations [4]. Cases with SMARCB1/INI1 loss have also been reported in tumors with mesenchymal and epithelial origins [2]. The present case outlines the inactivation of INI-1 resulting in renal medullary carcinoma of a young adult with very aggressive behavior.

## Case presentation

**Figure 1:**
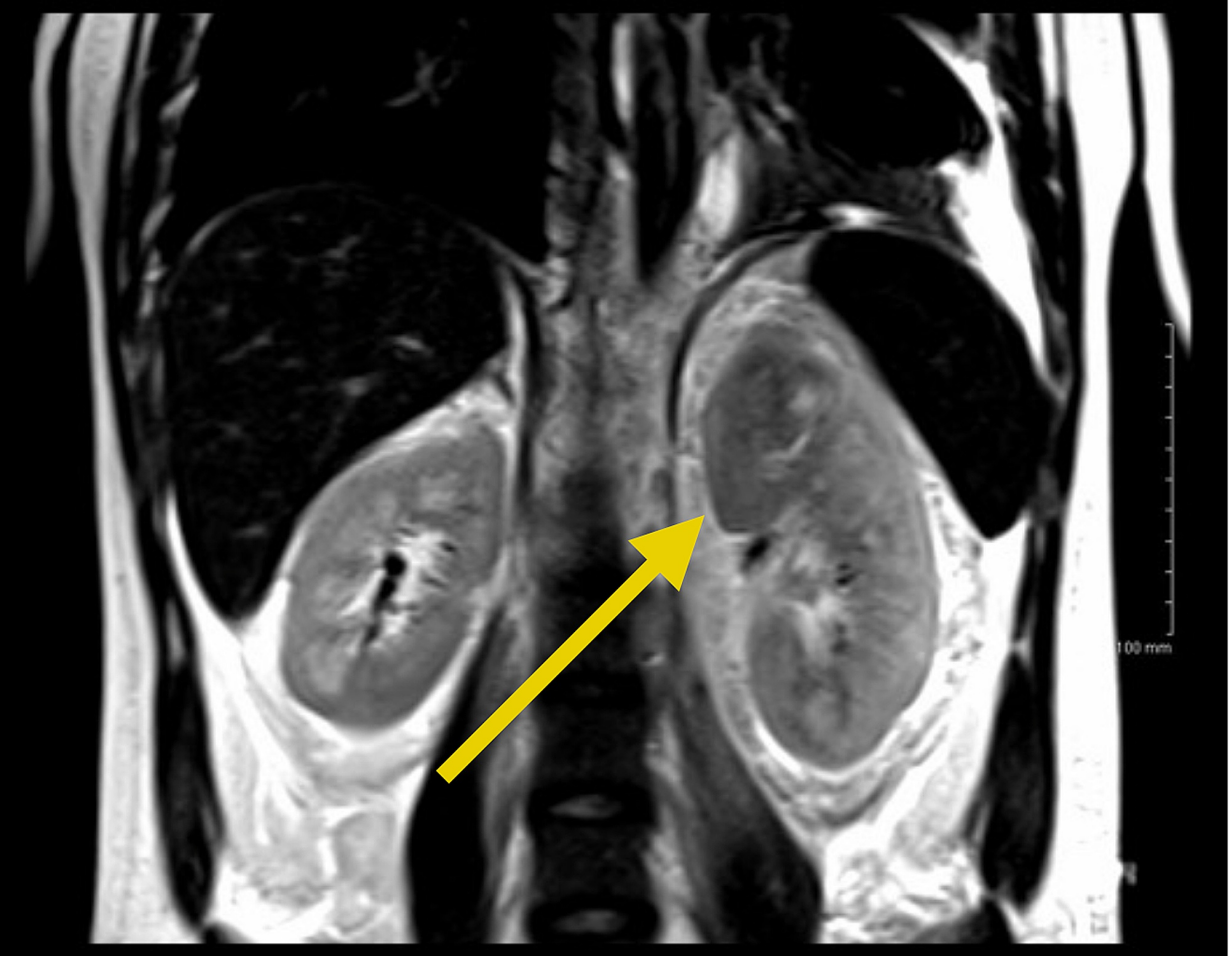
Left superior renal mass

**Figure 2:**
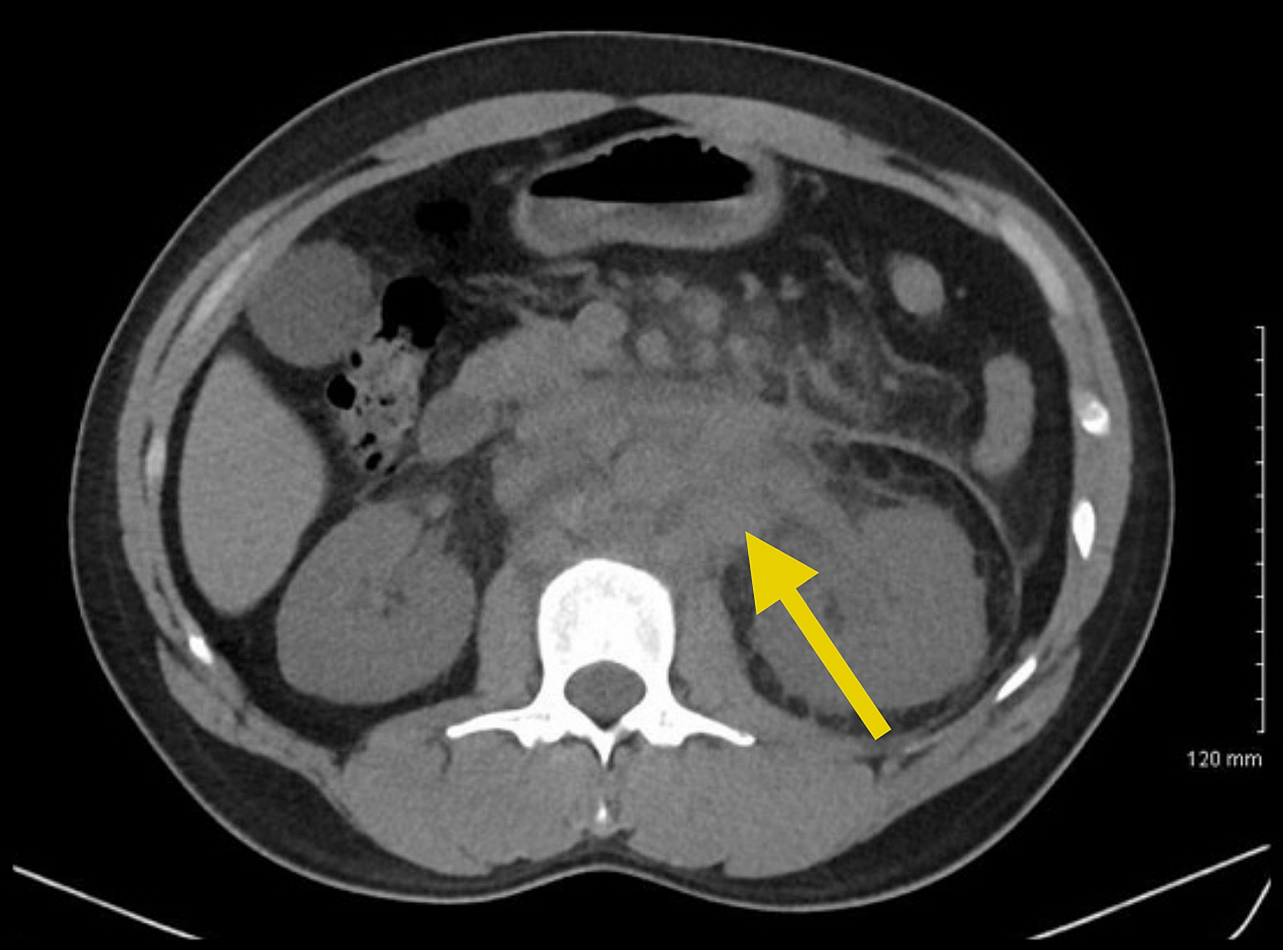
Diffuse retroperitoneal lymphadenopathy

A previously healthy 29-year-old Asian man presented to our emergency room with severe abdominal pain, hematuria, fevers, and chills. Computed tomography (CT) of the abdomen revealed a large ill-defined right renal mass, with multiple enlarged retroperitoneal, mediastinal and supraclavicular lymph nodes (Figures 1-2). The patient did not have any prior or family history of malignancy. Left supraclavicular node biopsy was performed. Pathology showed a poorly differentiated sarcomatoid renal cancer with immunohistochemistry testing revealing a tumor that was lacking staining for the INI-1 protein (member of SWI/SNF family). Radiation therapy was initiated for pain related to rapidly growing retroperitoneal lymphadenopathy. He received immunotherapy with a combination of ipilimumab and nivolumab within a week of diagnosis. Genetic testing revealed a heterozygous deletion of the gene encoding for the subunit SMARCA4 (c.665C>T). His clinical course continued to deteriorate rapidly. He developed an additional metastatic disease over the next few weeks and died of disease progression within eight weeks of diagnosis.

**Figure 3:**
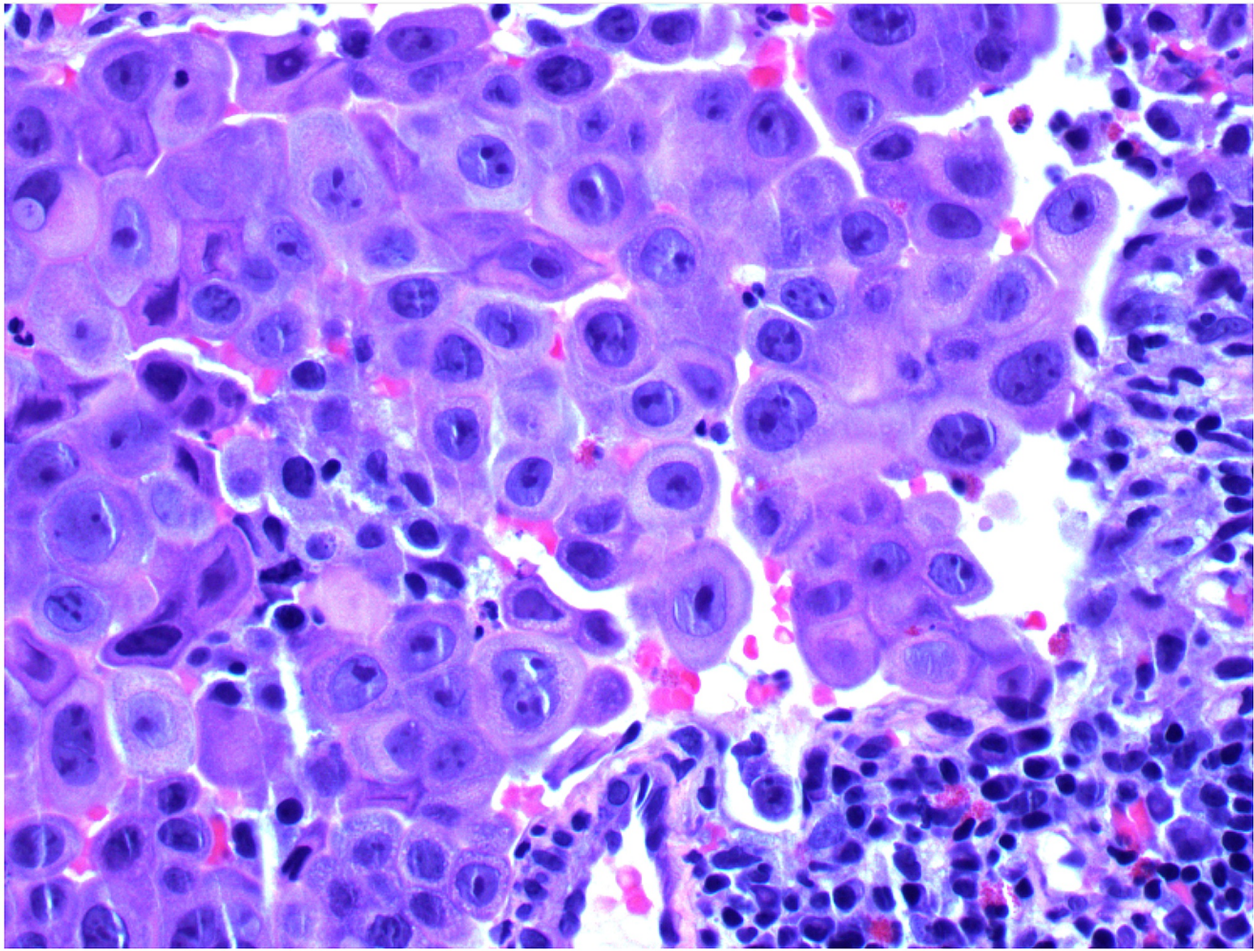
Tumor cells exhibiting rhabdoid cell features

The histologic diagnosis of MRT is seen based on the observation of medium to large cells with eccentrically located nuclei and abundant cytoplasm, often with paranuclear filamentous inclusions, and a polyphenotypic immunoprofile with frequent expression of vimentin, keratin, and epithelial membrane antigen (Figure 3) [5].

## Discussion

MRTs are a rare but devastating subtype of renal medullary carcinoma that is at times associated with patients with sickle cell disease or trait [5]. INI-1 protein, member of the SWI/SNF gene complex plays an important role in chromatin modification in an ATP-dependent manner [2]. INI-1’s loss of expression in mice plays a role in the development of rhabdoid tumors and aggressive lymphomas with nearly 100% mortality [1]. Deletions of 22q11.2 were first identified in MRT of childhood, and led to the identification of the SWI/SNF gene complex as an important tumor suppressor gene [2].

Biegel et al. studied 18 patients with atypical teratoid rhabdoid tumors (ATRT) of the brain, seven patients with renal MRTs, and four patients with extrarenal rhabdoid tumors [6]. All patients had INI-1 gene abnormalities; four of the seven with renal MRTs revealing germline mutations of the INI-1 gene. In another study, pediatric patients who were diagnosed with ATRTs and non-central nervous system MRTs were found to exhibit heterozygous germline INI-1 abnormalities [6]. In a study involving mice, researchers found that homozygous knockout of the INI-1 gene resulted in embryonic death, while heterozygous knockout resulted in normal appearance at birth but was found to later develop tumors histologically similar to MRT in later life [7].

Currently, the pathogenesis is not fully understood but the high prevalence of SMARCB1/INI-1 protein loss has been examined in prior case studies and it has been proposed this deletion to be a critical molecular alteration that drives tumor development [5]. The SWI/SNF complex regulates chromatic remodeling and therefore controls gene expression. Somatic mutations of several genes in this complex have been identified in multiple cancers. SWI/SNF gene complex is inactivated homozygously in the majority of MRT by either mutations or deletions [8]. Staining for SMARCB1/INI-1 (an important component of this protein complex) is absent in these tumors and has diagnostic value [5]. Uncommon germline mutations of members of this complex can cause a familial syndrome that includes childhood MRT, sarcomatoid renal tumors, and schwannomas [2]. The phenotypic rhabdoid appearance in renal cell tumors is evidence of potentially aggressive behavior [9]. The genetic connection between the histological rhabdoid appearance and the aggressive nature of the tumor is still uncertain. Our patient developed a sarcomatoid renal tumor as an adult and was found to have a heterozygous mutation of SMARCA4 (member of SWI/SNF complex). Typically, these patients present with rapidly progressive metastatic cancer that is usually refractory to standard oncological therapy.

## Conclusions

Somatic mutations in the SWI/SNF complex (SMARCA4, SMARCB1/INI-1) are common in many cancers. Germline mutations are uncommon, and usually cause a wide range of childhood cancers. These tumors have a highly aggressive course, and indeed, our patient died within weeks of his diagnosis. Additional research may help identify more appropriate therapies targeting this gene complex with may prove therapeutic value in patients with INI-1 deficient renal medullary carcinoma. 
